# Supplementary material for: Deciphering the Metabolic Basis and Molecular Circuitry of the Warburg Paradox in Lymphoma
Source: Cancers (Basel). 2024 Oct 25;16(21):3606. doi: 10.3390/cancers16213606 (PMC11545614; doi:10.3390/cancers16213606)

Deciphering the metabolic basis and molecular circuitry of the Warburg's pardox in lymphoma

Dashnamoorthy Ravi, Athena Kritharis, Andrew M. Evens

Supplementary Figures and Tables.

Figure S1. Pharmacological inhibition of molecular-metabolic Warburg circuitry. Graphs show the effect of pharmacological inhibitors on cell proliferation measured by MTT based cell viability assays in LCL, CA46, and SUDHL4 cells after 72 hours. X-axis represents the concentration of the pharmacological inhibitors, and Y-axis represents percent control cell viability. The error bars represent standard deviations of mean from experiments performed in triplicate.

Figure S2. Fractional labeling patterns in citric acid cycle intermediates and nucleotide metabolism with inhibitors of transcriptional regulators. Bar graphs represents mean fractional labeling patterns in **(a)** the citric acid cycle and **(b)** nucleotides, following $^{13}C_{1,2}$-Glucose isotope tracer (**c)** citric acid cycle following $^{13}C_{5,}$$^{15}N_2$-Glutamine isotope tracer labeling in LCL, CA46 and SUDHL4 treated with inhibitors. Error bars represent standard deviations from the mean of experimental triplicates. **(d)** Bar graphs represent relative carbon contributions in the pentose phosphate pathway and the effect of fludarabine treatment on carbon contributions to oxidative pentose phosphate pathway in lymphoma and LCL cells.

Figure S3. Western blot original scans relevant to Figure 8c

Table S1. Metabolic profiling raw data tables from all experiments. (attached as Table)

Table S2. Log_2 transformed RNA expression datasets corresponding to molecular and metabolic regulators of Warburg metabolism from Diffuse Large B Cell Lymphoma patient (NCI dataset) and lymphoma cell lines. (attached as Table)

Figure S1

a) Metabolic circuitry inhibitors

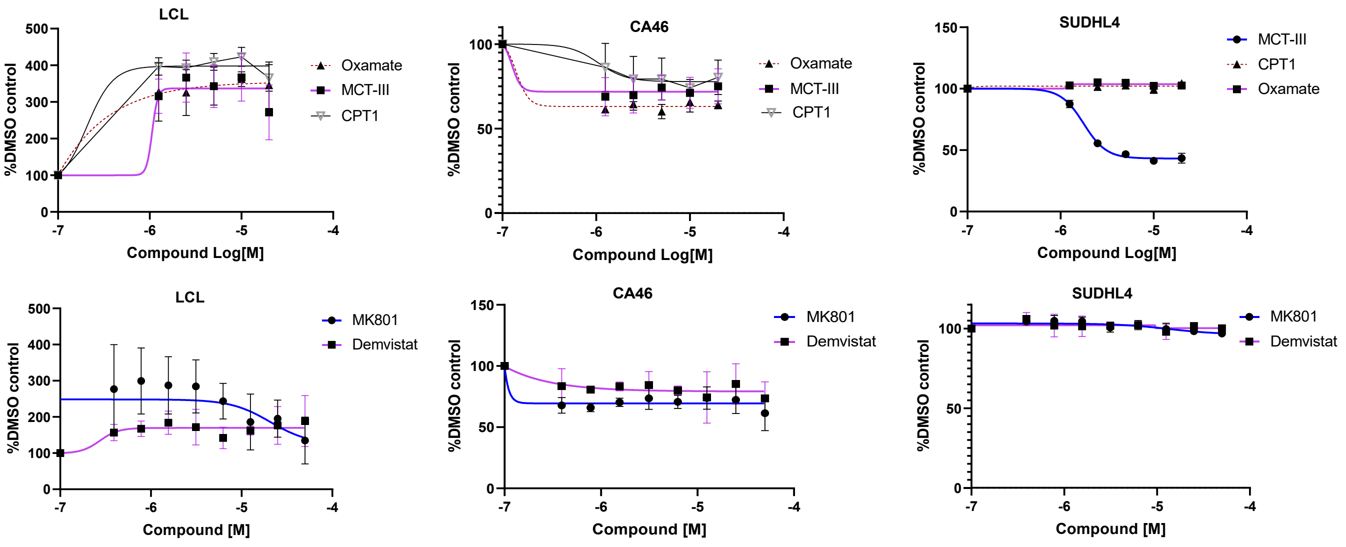

b) Molecular circuitry inhibitors

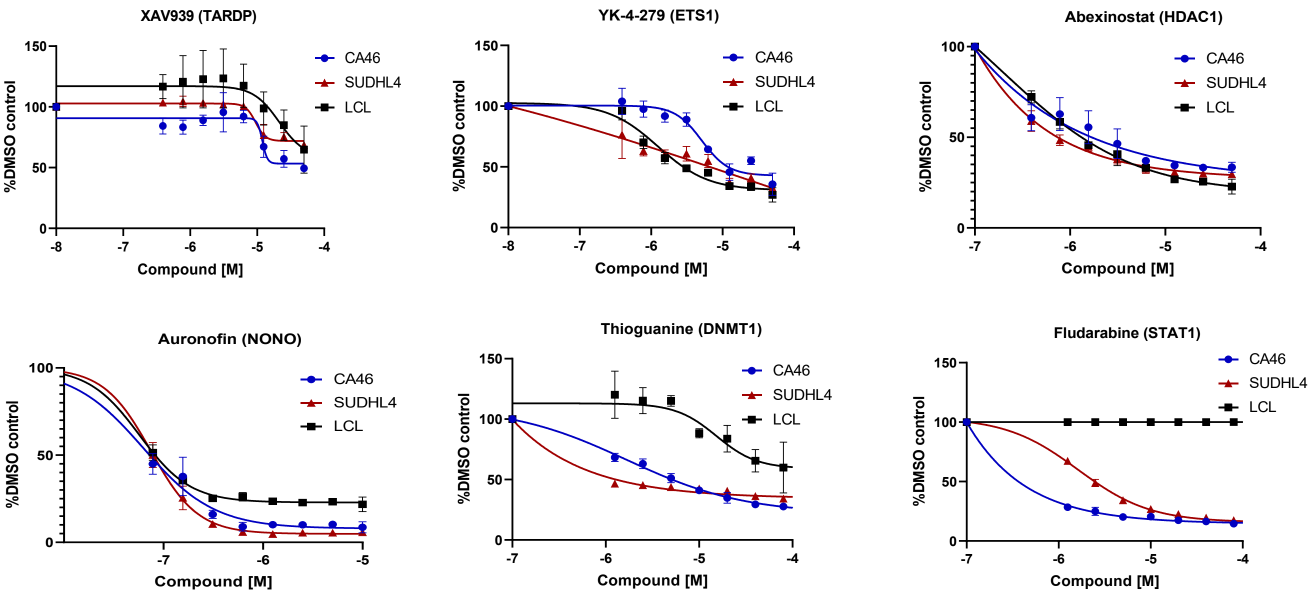

Figure S1. Pharmacological inhibition of molecular-metabolic Warburg circuitry. Graphs show the effect of pharmacological inhibitors on cell proliferation measured by MTT based cell viability assays in LCL, CA46, and SUDHL4 cells after 72 hours. X-axis represents the concentration of the pharmacological inhibitors, and Y-axis represents percent control cell viability. The error bars represent standard deviations of mean from experiments performed in triplicate.

Figure S2

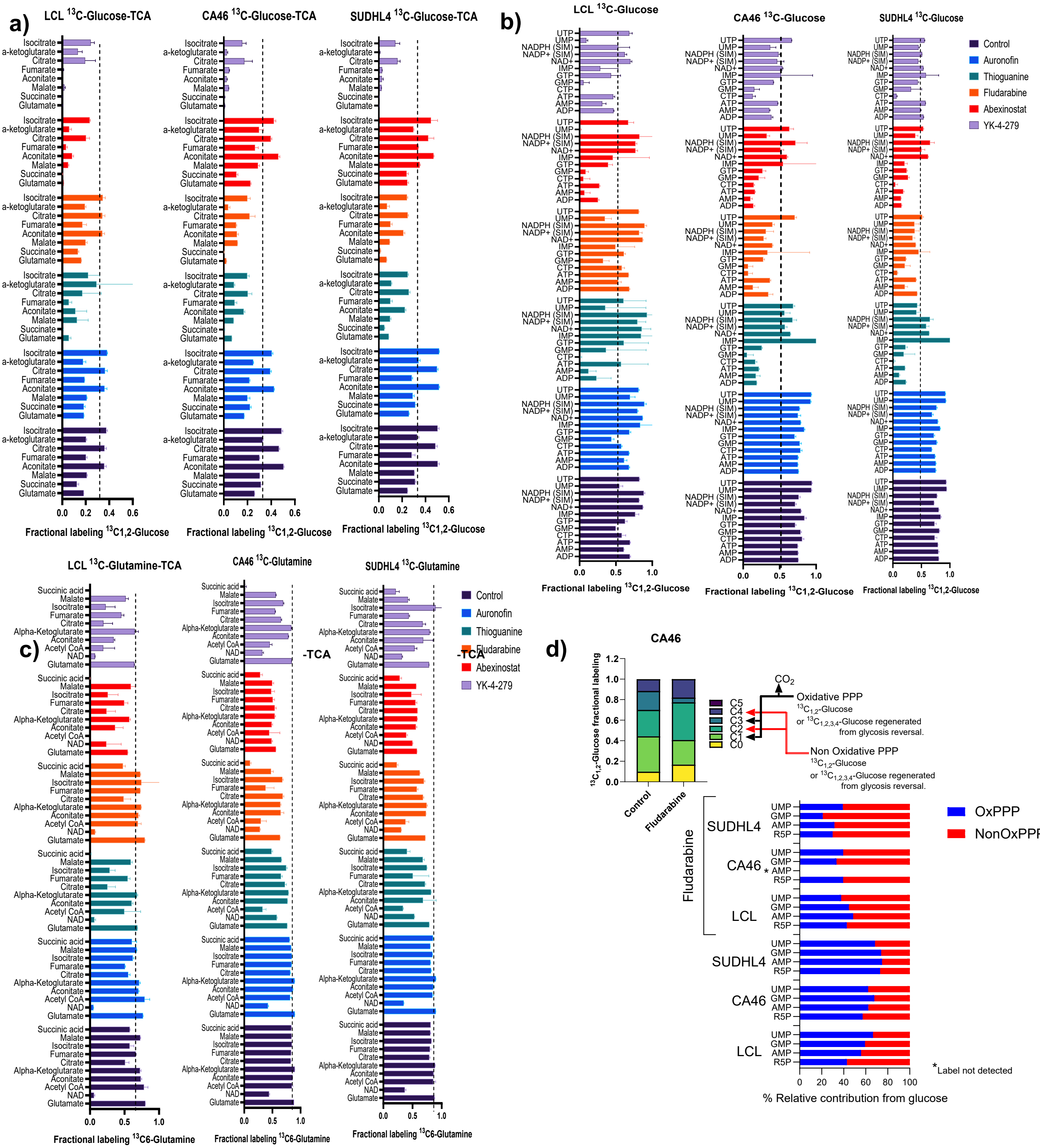

Figure S2. Fractional labeling patterns in citric acid cycle intermediates and nucleotide metabolism with inhibitors of transcriptional regulators. Bar graphs represents mean fractional labeling patterns in **(a)** the citric acid cycle and **(b)** nucleotides, following $^{13}C_{1,2}$-Glucose isotope tracer (**c**) citric acid cycle following $^{13}C_5$,$^{15}N_2$-Glutamine isotope tracer labeling in LCL, CA46 and SUDHL4 treated with inhibitors. Error bars represent standard deviations from the mean of experimental triplicates. **(d)** Bar graphs represent relative carbon contributions in the pentose phosphate pathway and the effect of fludarabine treatment on carbon contributions to oxidative pentose phosphate pathway in lymphoma and LCL cells.

Figures S3 (a-l)

Western blot original scans for Figure 8c

Figure S3a

STAT1

Data represented in Figure 8c is indicated within red box

Includes multiple exposures from Biorad Chemidoc

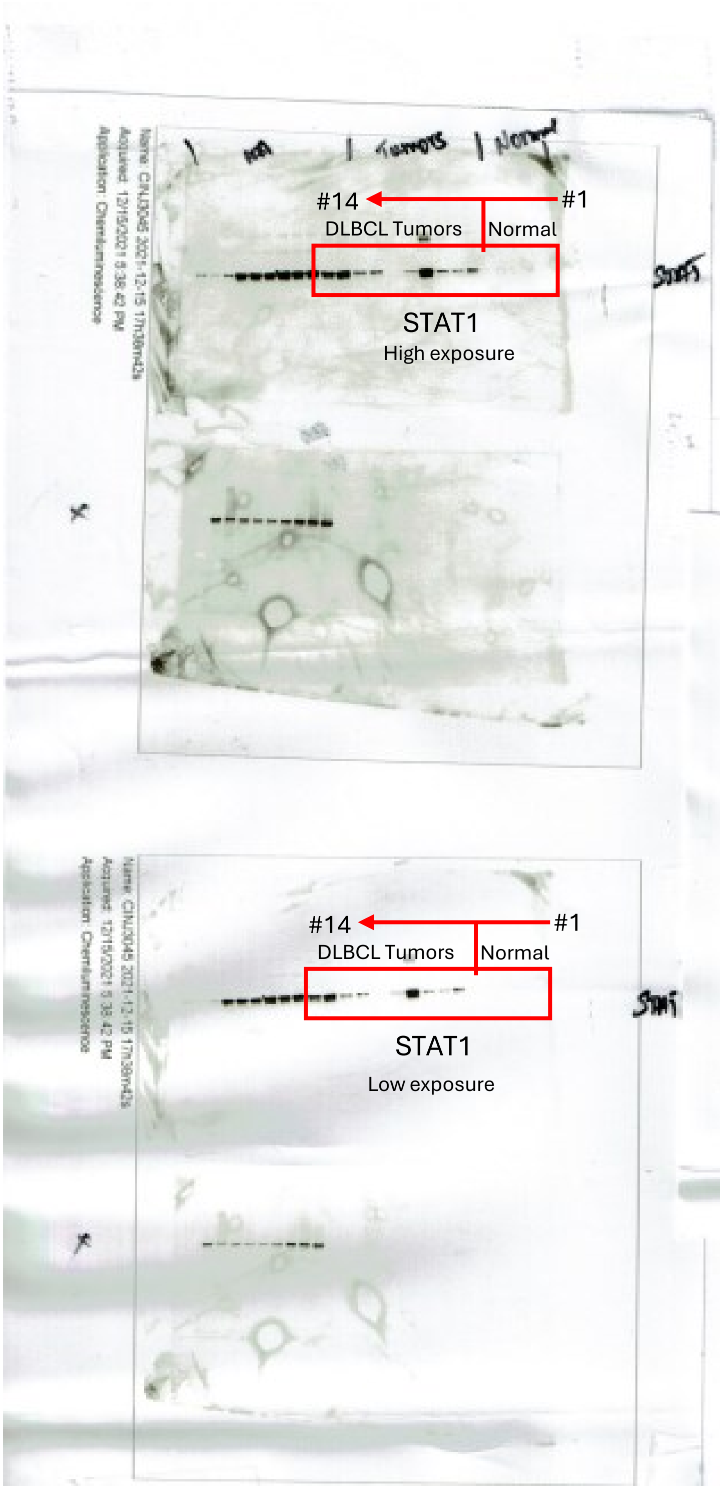

Image of the plain membrane

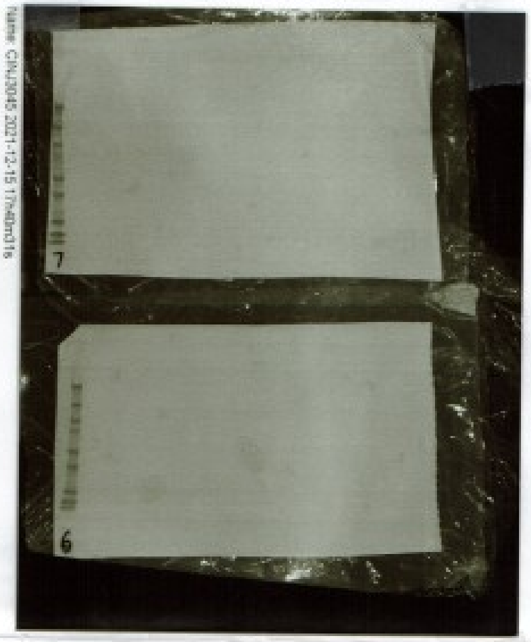

Figure S3b

HDAC1 & Jun

Data represented in Figure 8c is indicated within red box

Includes multiple exposures from Biorad Chemidoc

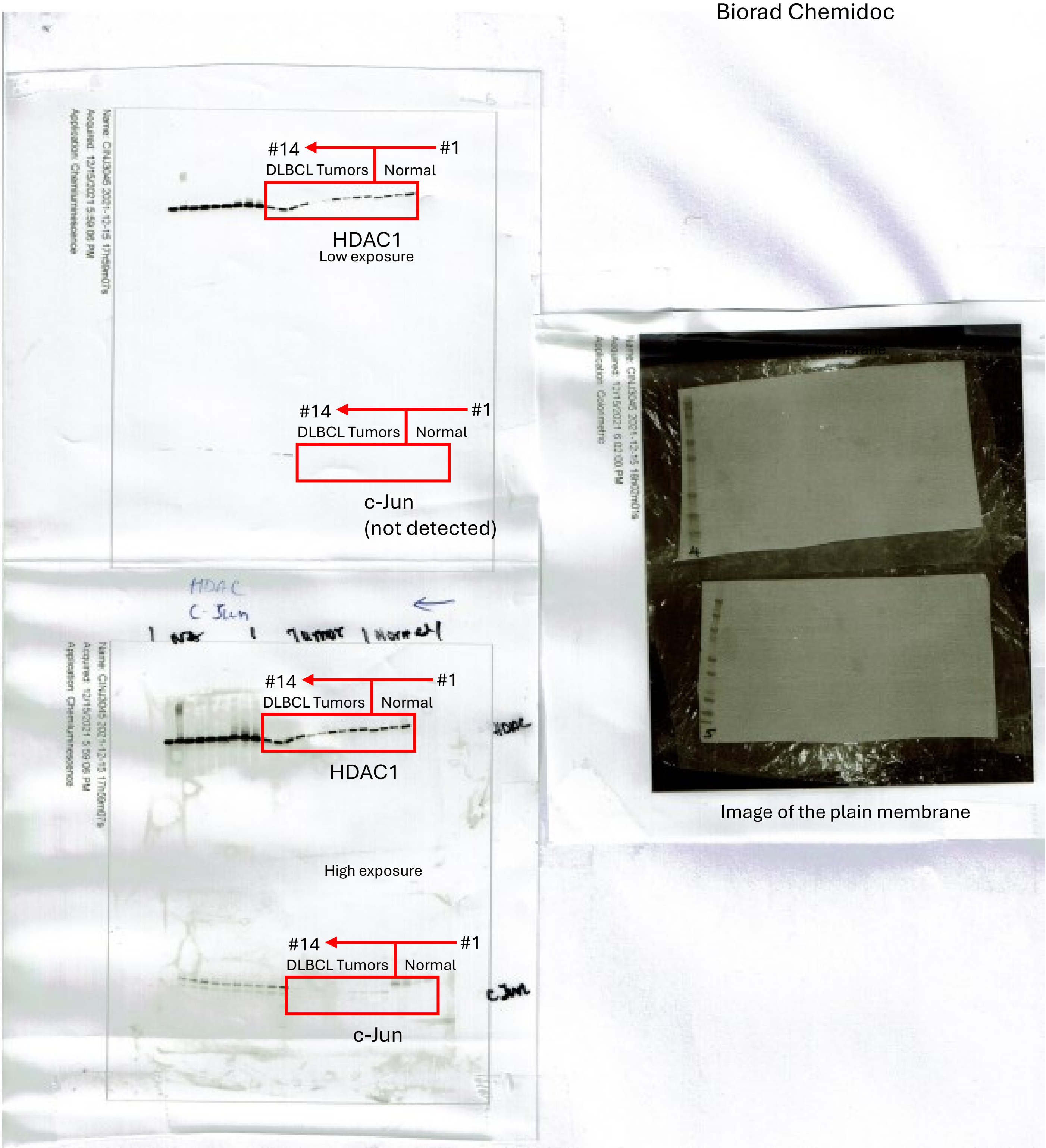

Figure S3c

HDAC1 & Jun
Additional

Data represented in Figure 8c is indicated within red box

Film exposure using the blot from previous page

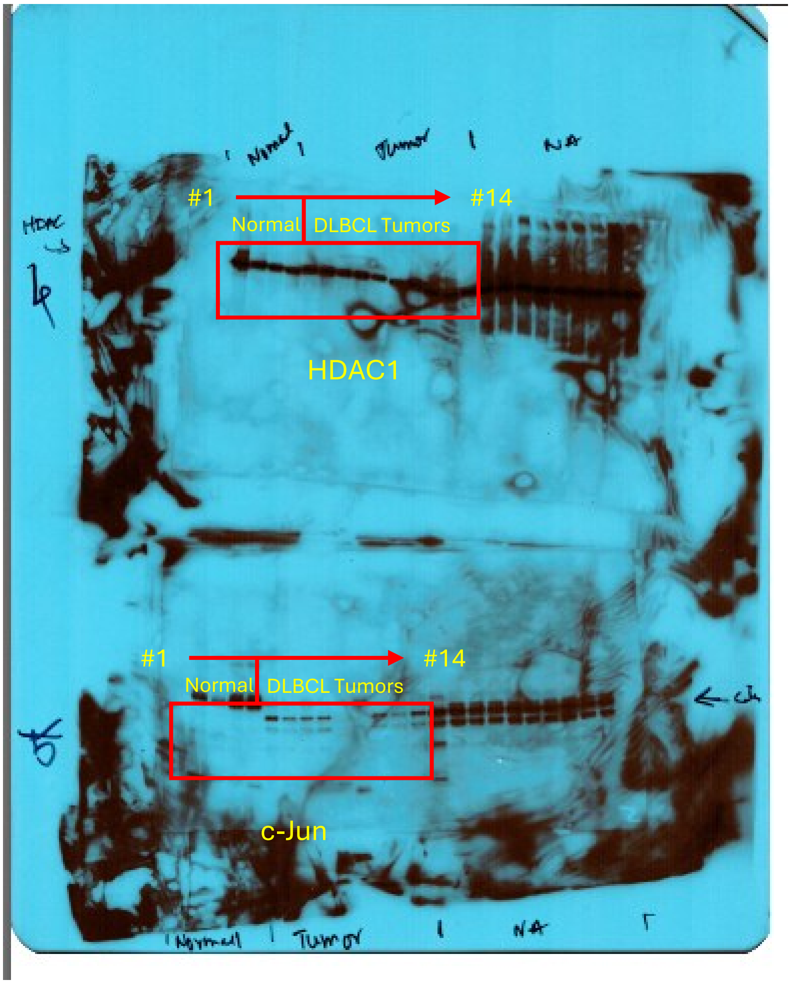

Figure S3d

PCNA

Data represented in Figure 8c is indicated within red box

Includes multiple exposures from Biorad Chemidoc

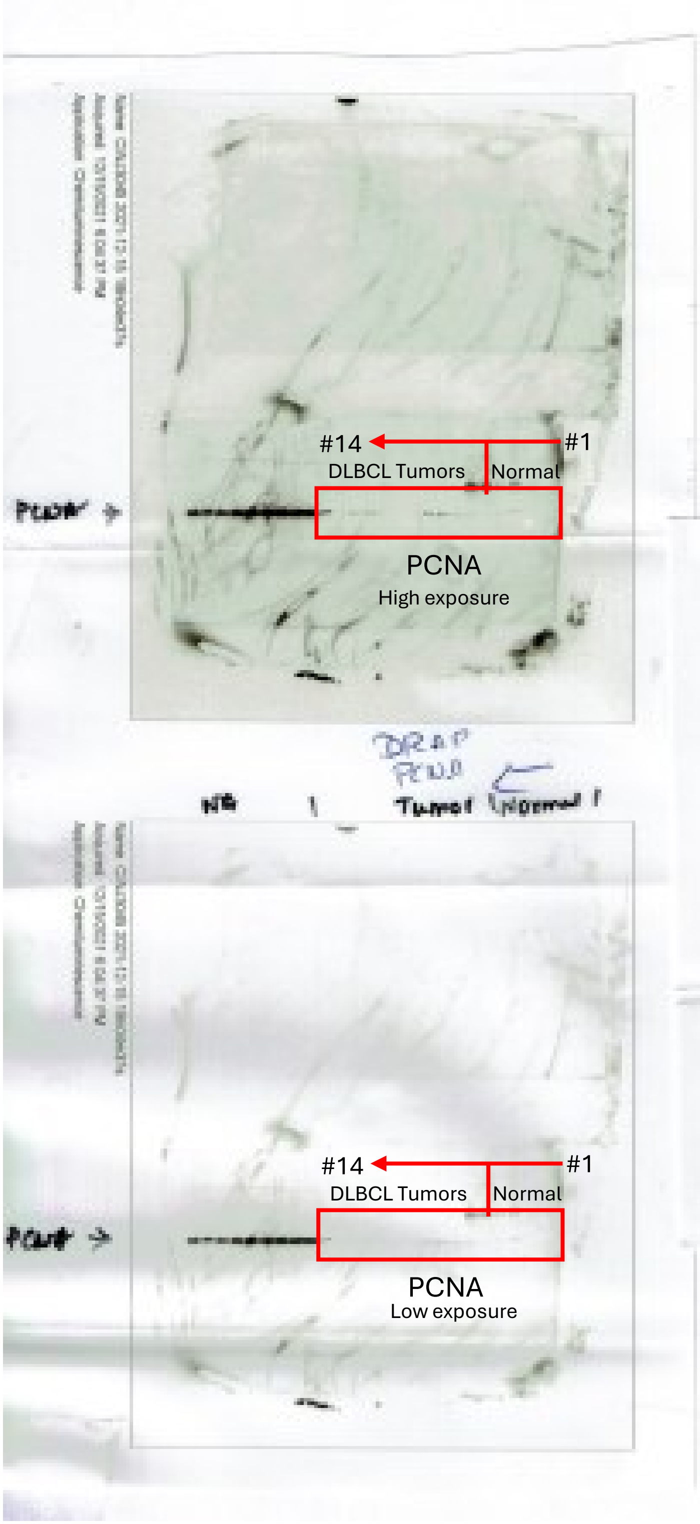

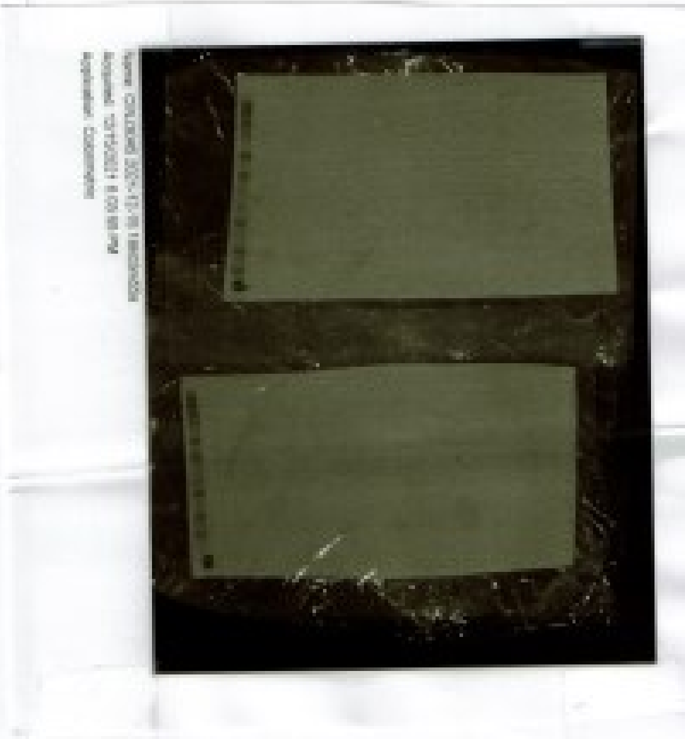

Figure S3e

PDHA

Data represented in Figure 8c is indicated within red box

Includes multiple exposures from Biorad Chemidoc

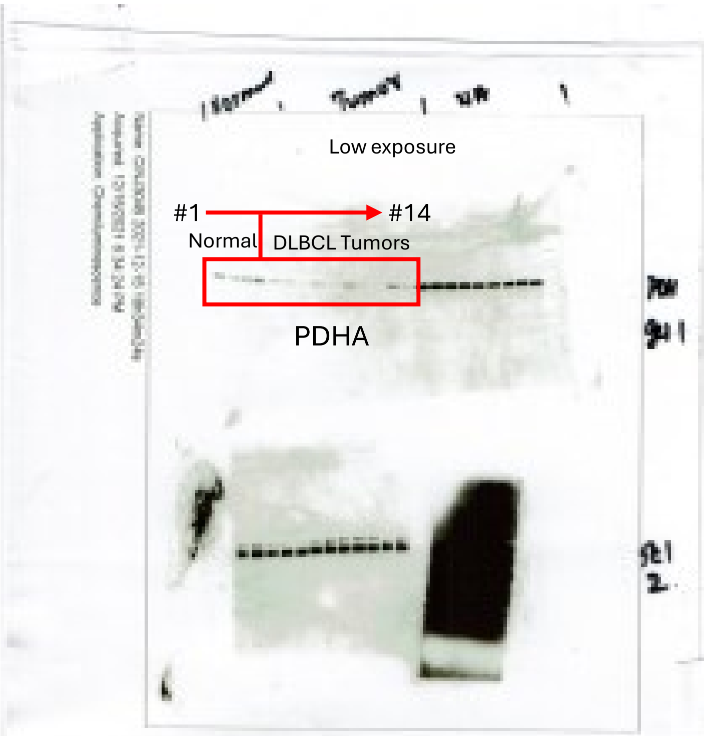

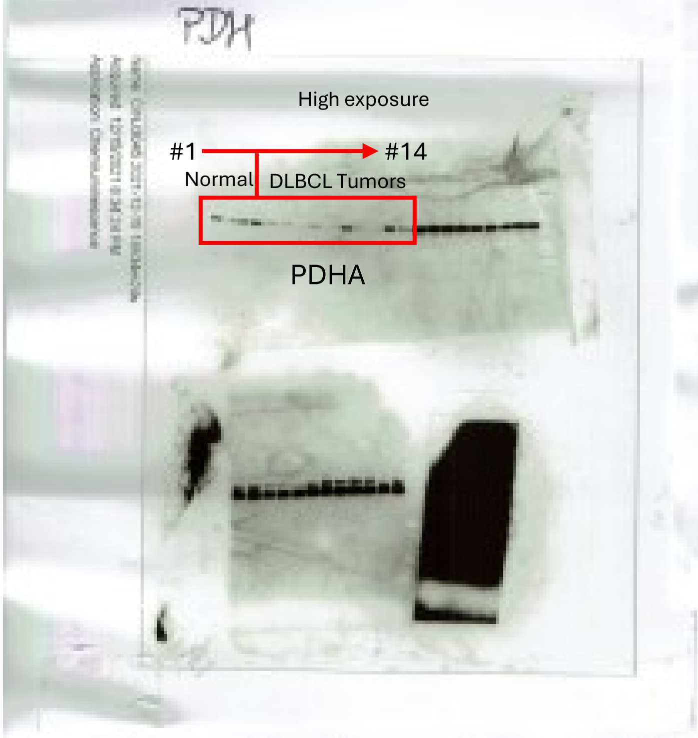

Image of the plain membrane

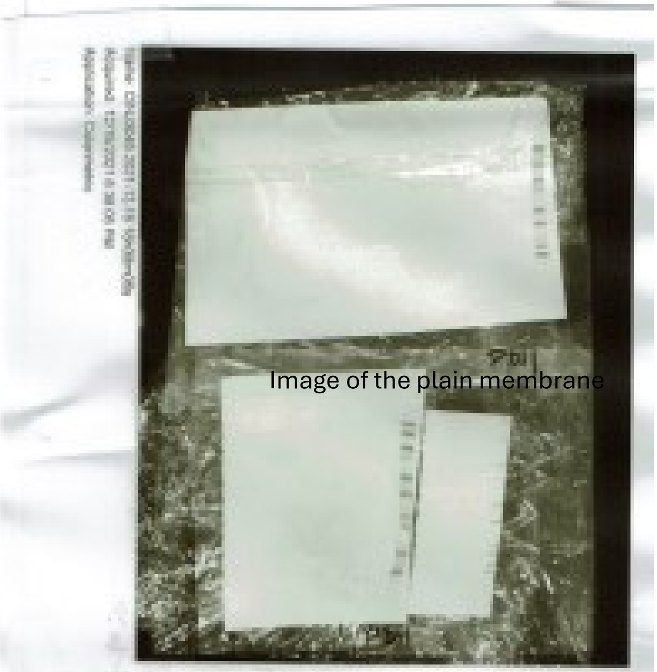

Figure S3f

PDHA

Data represented in Figure 8c is indicated within red box

Film exposure using the blot from previous page

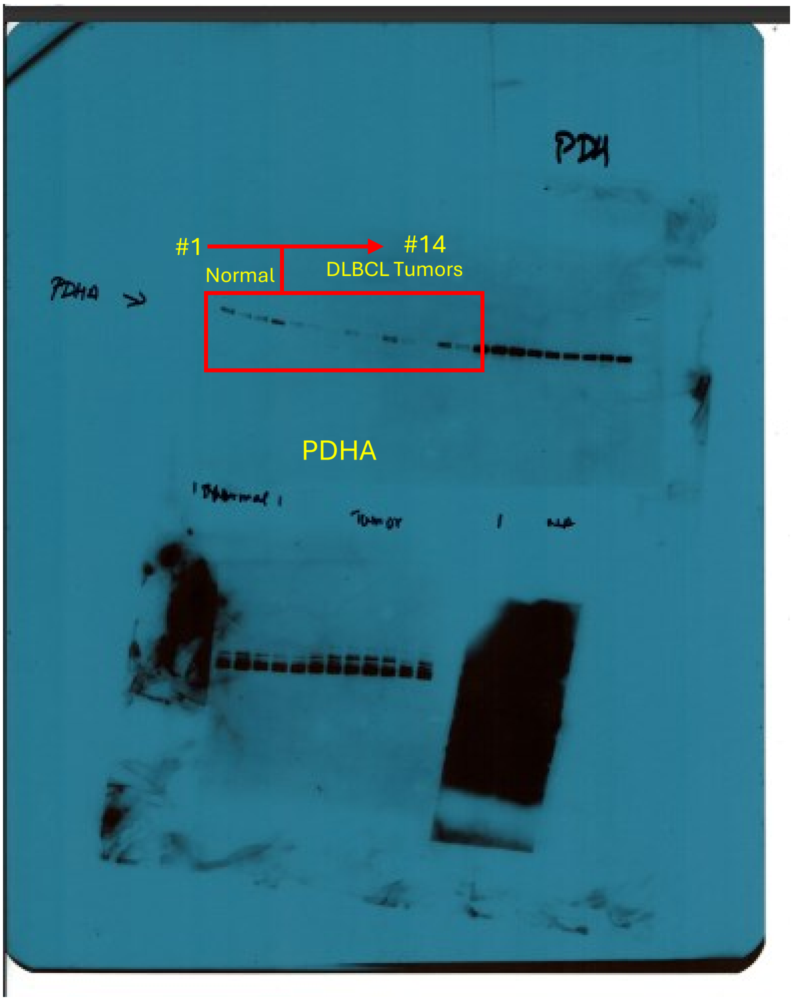

Figure S3g

LDHA

Data represented in Figure 8c is indicated within red box

Includes multiple exposures from Biorad Chemidoc

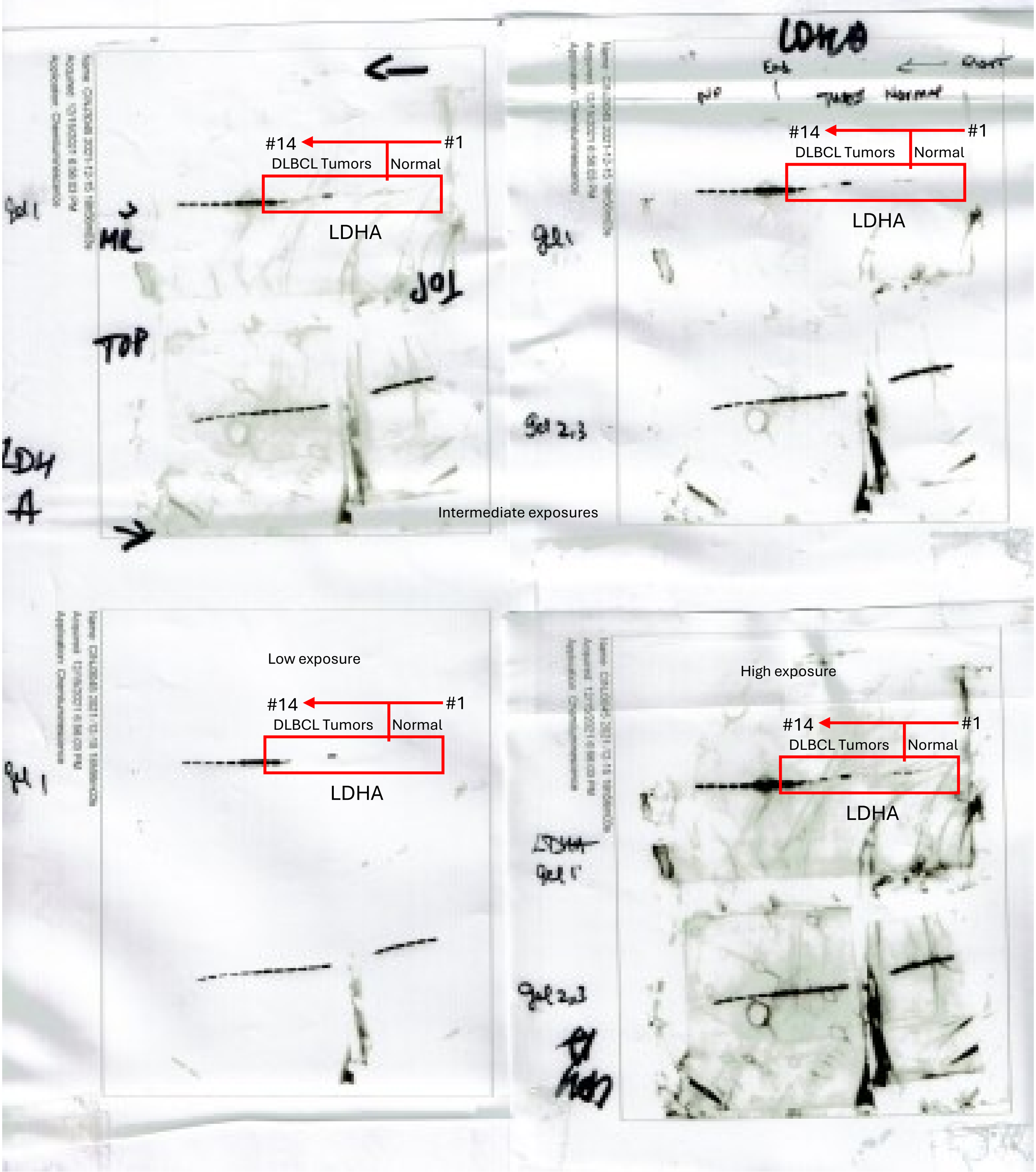

Figure S3h

LDHB

Data represented in Figure 8c is indicated within red box

Includes multiple exposures from Biorad Chemidoc

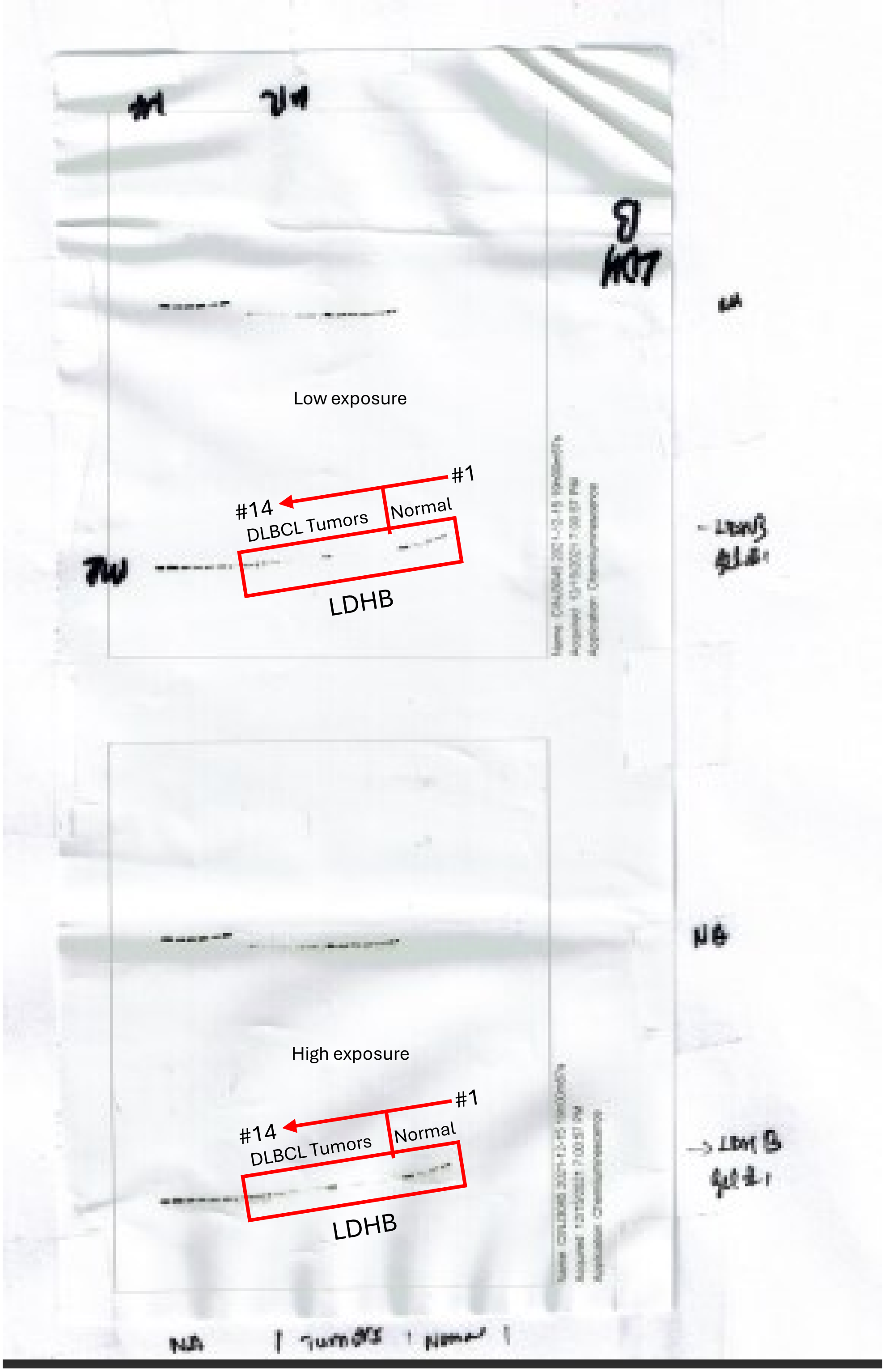

Figure S3i

ALT

Data represented in Figure 8c is indicated within red box

Includes multiple exposures from Biorad Chemidoc

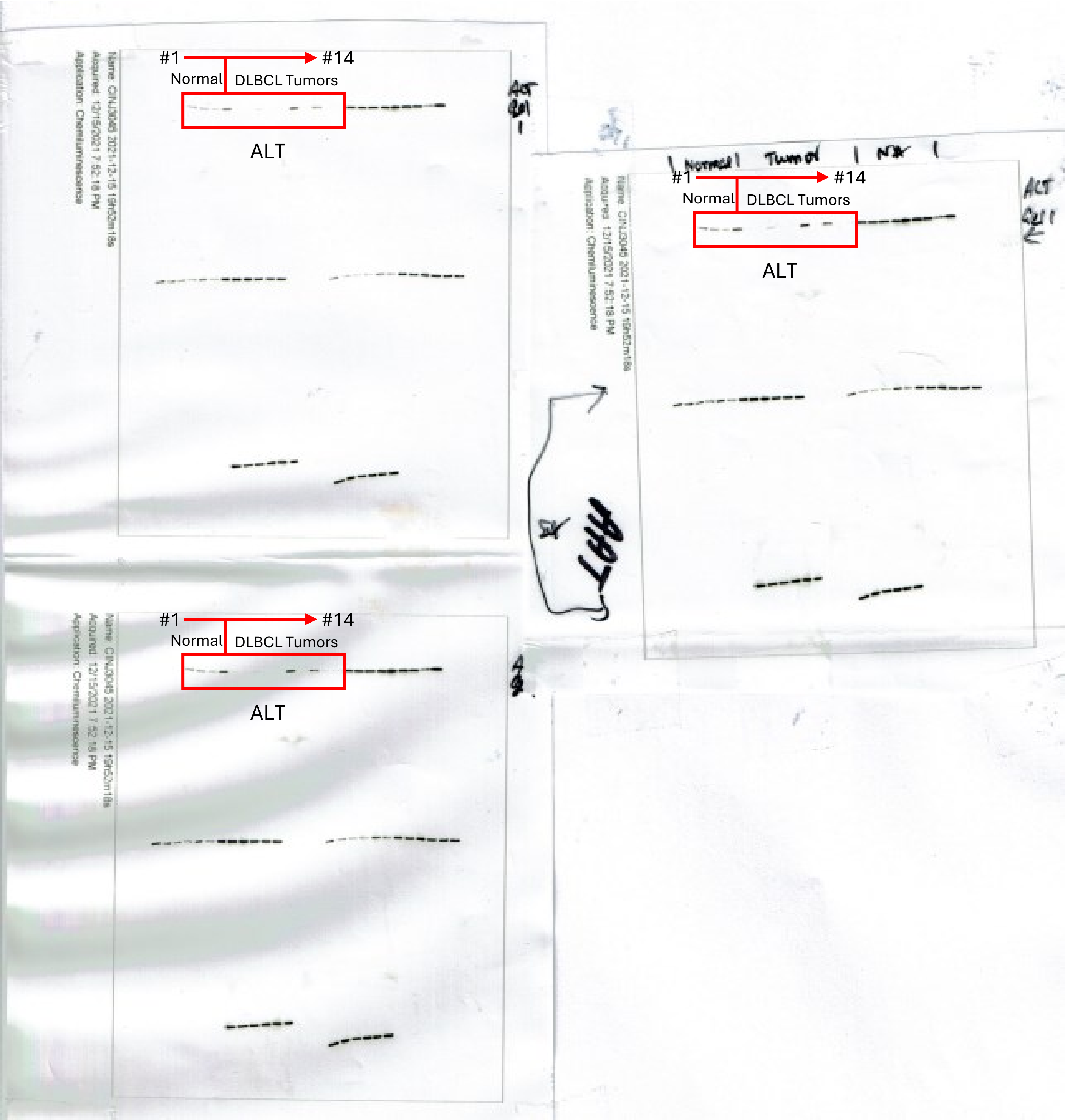

Figure S3j

GPT1

Data represented in Figure 8c is indicated within red box

Includes multiple exposures from Biorad Chemidoc

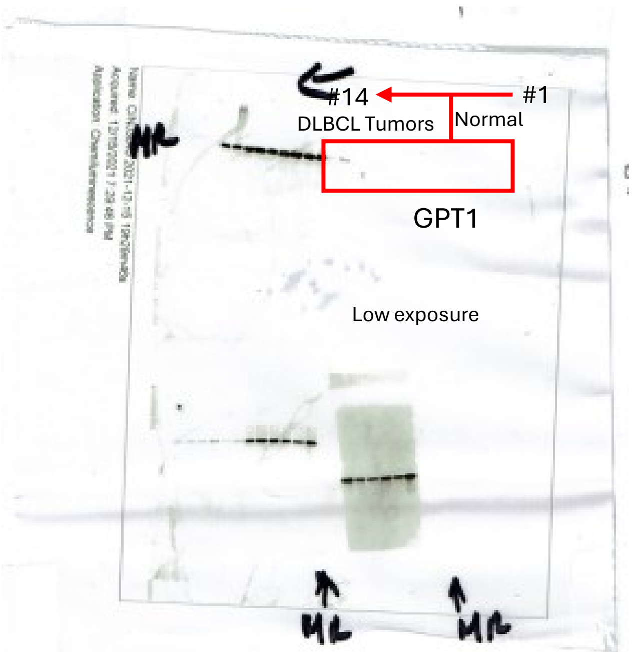

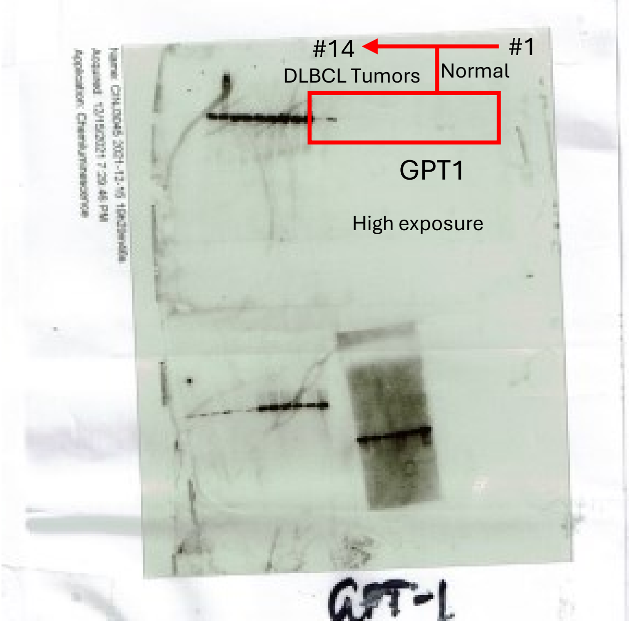

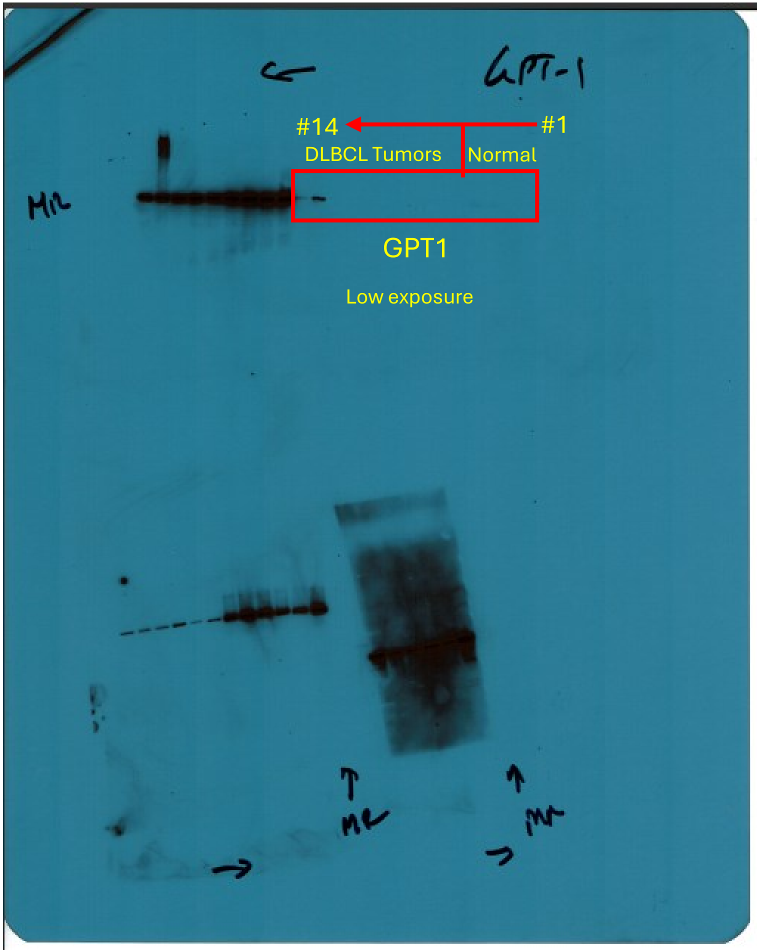

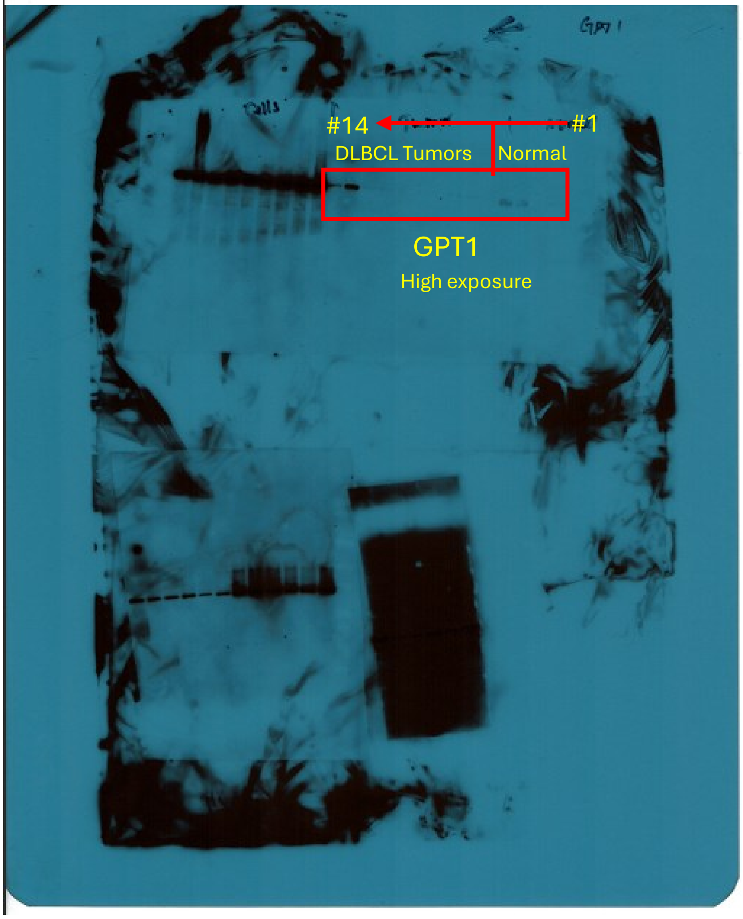

Figure S3k
β-actin

Data represented in Figure 8c is indicated within red box

Includes multiple exposures from Biorad Chemidoc

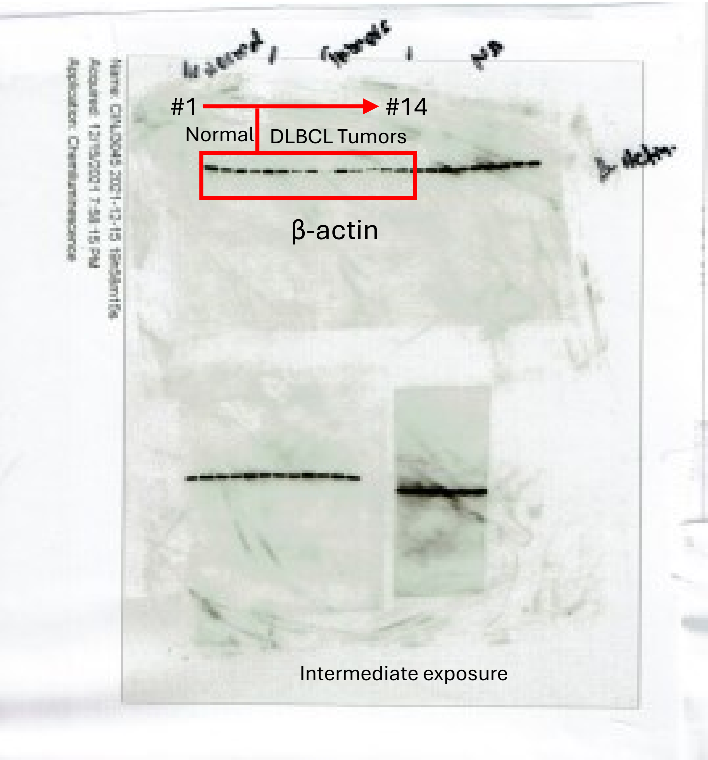

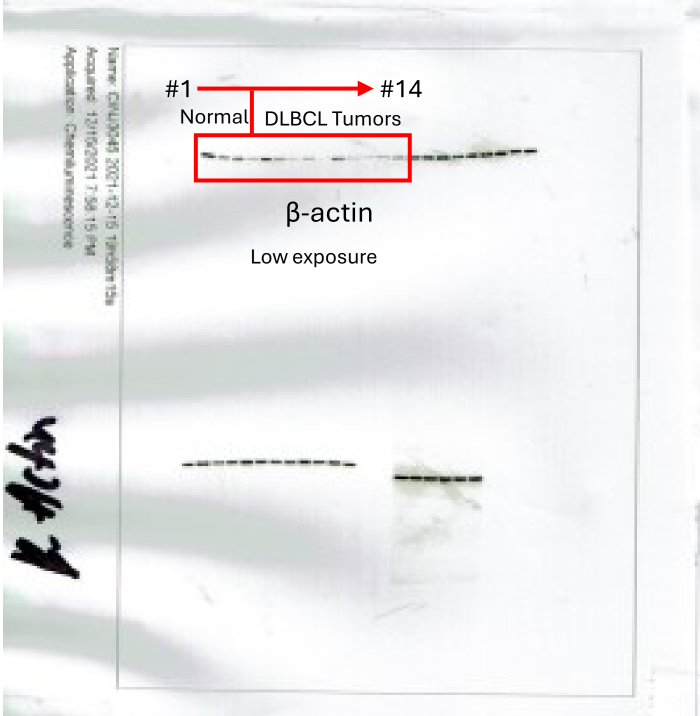

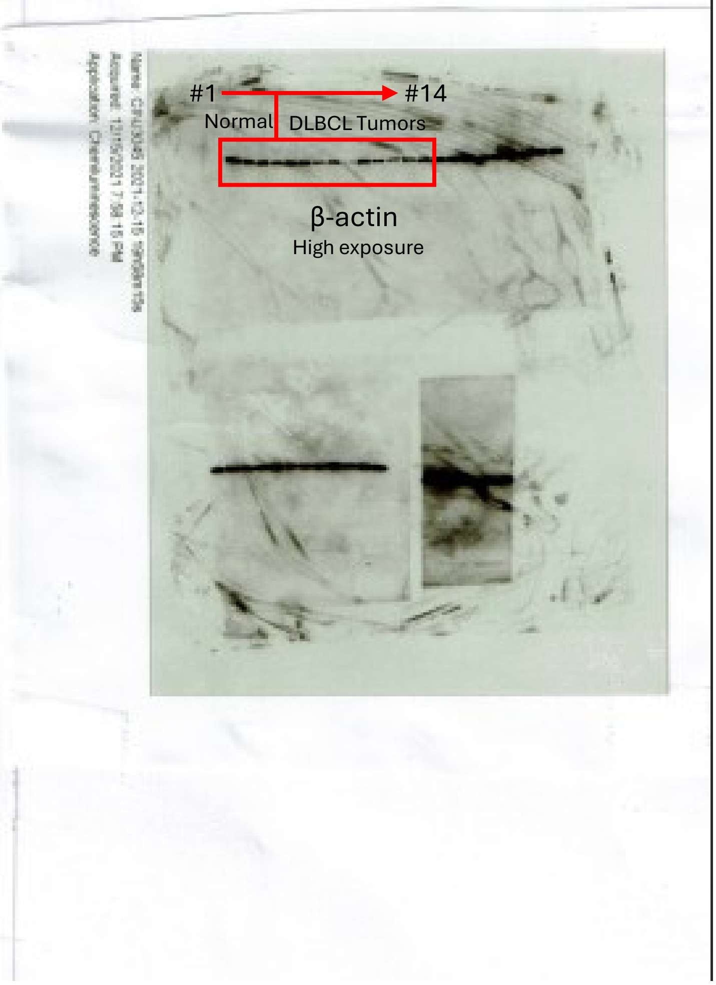

Figure S3I

β-actin

Data represented in Figure 8c is indicated within red box

Film exposure using the blot from previous page

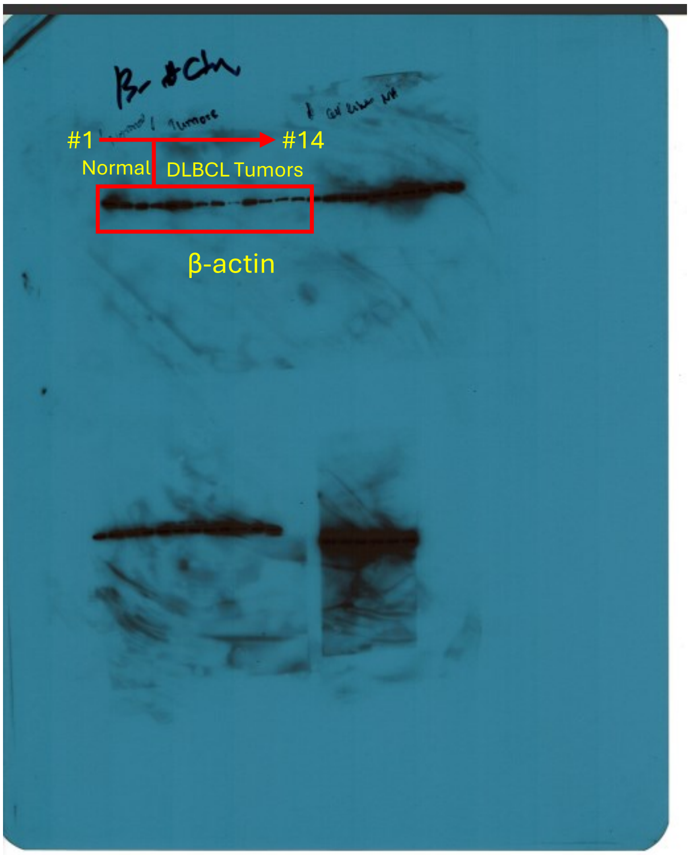

Supplement: Supplementary file 1 [file cancers-16-03606-s001.zip › Supplementary Figures and Legends.pdf]
